# Tumor-Homing pH-Sensitive Extracellular Vesicles for Targeting Heterogeneous Tumors

**DOI:** 10.3390/pharmaceutics12040372

**Published:** 2020-04-17

**Authors:** Jaeduk Park, Hyuk Lee, Yu Seok Youn, Kyung Taek Oh, Eun Seong Lee

**Affiliations:** 1 Department of Biotechnology, The Catholic University of Korea, 43 Jibong-ro, Bucheon-si, Gyeonggi-do 14662, Korea; jduck0309@naver.com (J.P.); ahld1421@naver.com (H.L.); 2 School of Pharmacy, Sungkyunkwan University, 2066 Seobu-ro, Jangan-gu, Suwon, Gyeonggi-do 16419, Korea; ysyoun@skku.edu; 3 College of Pharmacy, Chung-Ang University, 221 Heukseok dong, Dongjak-gu, Seoul 06974, Korea; kyungoh@cau.ac.kr; 4 Department of Biomedical Chemical Engineering, The Catholic University of Korea, 43 Jibong-ro, Bucheon-si, Gyeonggi-do 14662, Korea

**Keywords:** tumor-homing extracellular vesicles, pH-sensitive extracellular vesicles, doxorubicin, tumor therapy

## Abstract

In this study, we fabricated tumor-homing pH-sensitive extracellular vesicles for efficient tumor treatment. These vesicles were prepared using extracellular vesicles (EVs; BTEVs extracted from BT-474 tumor cells or SKEVs extracted from SK-N-MC tumor cells), hyaluronic acid grafted with 3-(diethylamino)propylamine (HDEA), and doxorubicin (DOX, as a model antitumor drug). Consequently, HDEA/DOX anchored EVs (HDEA@EVs) can interact with origin tumor cells owing to EVs’ homing ability to origin cells. Therefore, EV blends of HDEA@BTEVs and HDEA@SKEVs demonstrate highly increased cellular uptake in both BT-474 and SK-N-MC cells: HDEA@BTEVs for BT-474 tumor cells and HDEA@SKEVs for SK-N-MC tumor cells. Furthermore, the hydrophobic HDEA present in HDEA@EVs at pH 7.4 can switch to hydrophilic HDEA at pH 6.5 as a result of acidic pH-induced protonation of 3-(diethylamino)propylamine (DEAP) moieties, resulting in an acidic pH-activated EVs’ disruption, accelerated release of encapsulated DOX molecules, and highly increased cell cytotoxicity. However, EV blends containing pH-insensitive HA grafted with deoxycholic acid (HDOC) (HDOC@BTEVs and HDOC@SKEVs) showed less cell cytotoxicity for both BT-474 and SK-N-MC tumor cells, because they did not act on EVs’ disruption and the resulting DOX release. Consequently, the use of these tumor-homing pH-sensitive EV blends may result in effective targeted therapies for various tumor cells.

## 1. Introduction

Extracellular vesicles (EVs) are nanosized cellular vesicles released from various types of tumor cells [1,2,3,4,5]. To achieve quick and extensive intercellular communication between tumor cells, EVs are secreted out of the cells so that they can enter the recipient cells [4,5,6,7]. These EVs perform various biological functions, such as the disposal of cellular waste products, release of foreign invaders, control of gene expression, and activation of the immune system [8,9,10].

In addition, EVs intrinsically express various membrane proteins, cell adhesion molecules, and tumor specific ligands, thereby enabling the homing of EVs to origin cells [4,11,12,13,14,15,16]. These properties of EVs enable tumor-homing ability and render them potential candidates as tumor-recognizing drug carriers. In particular, recent studies suggest that these EVs have the ability to interact with their released parental cells, and this property has been used to target tumor cells [4,11,12,13,14,15,16]. This means that these EVs are suitable as tumor-targeting and tumor-penetrating drug carriers, as they can be selectively homed to their parent tumor cells [4,13,14,15,16,17,18,19,20,21,22]. Furthermore, the immunogenicity of these EVs is relatively low; therefore, they exhibit excellent body safety for biomedical applications [4,10,19,20].

In this study, we fabricated tumor-homing pH-sensitive EVs. These EVs were prepared using EVs (BTEVs extracted from BT-474 tumor cells or SKEVs extracted from SK-N-MC tumor cells), hyaluronic acid grafted with 3-(diethylamino)propylamine (HDEA), and doxorubicin (DOX) [3]. In particular, we prepared EV blends using HDEA/DOX anchored EVs (HDEA@BTEVs and HDEA@SKEVs) to target different tumor cells. The EV blends are expected to yield efficient cellular uptake for parent BT-474 and SK-N-MC tumor cells. Therefore, we hypothesize that these different EVs can target their origin tumor cells, owing to their homing ability to origin cells, allowing their efficient accumulation into heterogeneous tumor cells. Furthermore, 3-(diethylamino)propylamine (DEAP) moieties present in HDEA can be protonated at endosomal pH and induce the destabilization of EVs, owing to DEAP-mediated vesicle destabilization, followed by the release of encapsulated DOX [3,23,24,25,26,27,28,29,30,31]. In this study, we investigated the tumor targeting ability, pH-sensitive properties, and antitumor efficacy of EV blends against BT-474 and SK-N-MC tumor cells.

## 2. Materials and Methods

### 2.1. Materials

Hyaluronic acid (HA, Mw = 4.8 kDa), 3-(diethylamino)propylamine (DEAP), N-hydroxysuccinimide (NHS), N,N’-dicyclohexylcarbodiimide (DCC), triethylamine (TEA), deoxycholic acid (DOCA), 4-dimethylaminopyridine (DMAP), pyridine, dimethyl sulfoxide (DMSO), sodium tetraborate, adipic acid dihydrazide (ADH), doxorubicin hydrochloride (DOX), paraformaldehyde, heparin, and Triton X-100, 4′,6-diamidino-2-phenylindole dihydrochloride (DAPI) were purchased from Sigma-Aldrich (St. Louis, MO, USA). Chlorin e6 (Ce6) was purchased from Frontier Scientific Inc (Logan, UT, USA). RPMI-1640 medium, DMEM medium, fetal bovine serum (FBS), phosphate buffered saline (PBS), ethylene diamine tetra-acetic acid (EDTA), penicillin, trypsin, and streptomycin were purchased from Welgene Inc (Seoul, Korea). EV-depleted FBS was purchased from System Biosciences Inc. (Palo Alto, CA, USA). Cell Counting Kit-8 (CCK-8) was purchased from Dojindo Molecular Technologies Inc. (Santa Clara, CA, USA). Wheat Germ Agglutinin Alexa Fluor^®^ 488 Conjugate (WGA-Alexa Fluor^®^ 488), fluorescein isothiocyanate (FITC) were purchased from Life Technologies (Carlsbad, CA, USA).

### 2.2. Synthesis of HA-g-DEAP

The detailed synthesis method of HA grafted with DEAP (HA-g-DEAP: HDEA) was described in our previous report [3,29,30,31]. Briefly, HA (200 mg) was reacted with DEAP (55 mg) in DMSO (10 mL) containing DCC (110 mg), NHS (60 mg), and TEA (500 µL) at 25 °C for 3 days, to produce HDEA (Figure S1). HA grafted with DOCA (HA-g-DOCA: HDOC) was prepared as a pH-insensitive control group against pH-sensitive HDEA. The detailed synthesis method of HDOC was described in our previous report [3,29,30,31]. Briefly, HA (200 mg) was reacted with DOCA (650 mg) in DMSO (10 mL) containing DCC (340 mg), DMAP (20 mg), and pyridine (500 µL) at 25 °C for 3 days, to produce HDOC (Figure S2). In addition, HDEA (100 mg) or HDOC (100 mg) were reacted with Ce6 (15 mg) in DMSO (10 mL) containing DCC (10 mg), ADH (9 mg), NHS (6 mg), and TEA (200 µL) at 25 °C for 2 days, to produce Ce6-labeled HDEA or Ce6–labeled HDOC [3,30,31]. The non-reacted chemicals were removed via dialysis against fresh DMSO for 3 days, and then deionized water for 3 days using a pre-swollen dialysis membrane (Spectra/Por^®^6 MWCO 2 kDa, Spectrum Laboratories Inc, Rancho Dominguez, CA, USA). The dialyzed solution was freeze-dried; subsequently, the final product was obtained [3,29,30,31].

### 2.3. Harvest of EVs

To harvest EVs, human breast carcinoma BT-474 cells and human neuroblastoma SK-N-MC cells were selected as the EV secreting cell lines and purchased from the Korean Cell Line Bank (Seoul, Korea). BT-474 cells were cultured in an RPMI-1640 medium containing 1% penicillin-streptomycin and 10% EV-depleted FBS in a 5% CO_2_ atmosphere at 37 °C. SK-N-MC cells were cultured in the DMEM medium containing 1% penicillin-streptomycin and 10% EV-depleted FBS in a 5% CO_2_ atmosphere at 37 °C. The cell culture medium was centrifuged at 2000 *g* for 20 min at 4 °C and additionally centrifuged at 10,000 *g* for 30 min to eliminate large cell debris and dead cells. Subsequently, the supernatant was again ultracentrifuged at 100,000 *g* at 4 °C for 70 min to separate EVs pellets. The obtained pellets were washed using fresh PBS (150 mM, pH 7.4) and then ultracentrifuged at 100,000 *g* at 4 °C for 70 min. These purified EVs were stored at −80 °C, after being suspended in fresh PBS (150 mM, pH 7.4) [3,30,32,33,34]. In addition, the suspended EVs concentration was analyzed using Nanosight (LM10, Malvern Instruments, Malvern, UK) with NTA 2.3 software [3,35].

### 2.4. Preparation of EV Samples 

Sonication was performed to incorporate HDEA (or HDOC) and DOX to EVs [3,30,36]. HDEA (300 μg, or Ce6-labeled HDEA) or HDOC (200 μg, or Ce6-labeled HDOC) dissolved in DMSO (0.1 mL) containing DOX (400 μg) were mixed with EVs (200 μg) suspended in PBS (10 mL, 150 mM, pH 7.4) at 25 °C, and the solution was sonicated using a tip sonicator, vcx-130 with cv-18 (Sonics, Newtown, CT, USA) with a 30% amplitude for 30 s. This sonication process was repeated 6 times at 3 min intervals. The obtained solution was incubated at 37 °C for 60 min to recover the EVs. The filtration method using 0.22 μm membranes was performed to remove HDEA (or HDOC) or DOX aggregates. Ultracentrifugation at 100,000 *g* at 4 °C for 70 min was performed to remove free HDEA (or HDOC) and DOX to yield HDEA@EVs or HDOC@EVs [3,30,36]. In addition, DOX@EVs with DOX and without HDEA and HDOC were prepared following the same procedure as described above. 

### 2.5. Measurement of Loading Contents 

The concentration of HDEA or HDOC (with fluorescent Ce6 dye) in the EVs was measured using a fluorescence spectrofluorometer (RF-5301PC, Shimadzu, Kyoto, Japan) at λ_ex_ of 450 nm and λ_em_ of 670 nm using a DMSO/PBS solution (90/10 vol.%) [3,30,31]. The HDEA or HDOC loading content (%) was calculated as the weight percentage of HDEA or HDOC in the EVs. The concentration of DOX entrapped in the EVs was measured using a fluorescence spectrofluorometer at λ_ex_ of 470 nm and λ_em_ of 592 nm using a DMSO/PBS solution (90/10 vol.%). The DOX loading content (%) was calculated as the weight percentage of DOX in the EVs [3,30,31]. 

### 2.6. Characterization of EV Samples 

The particle size and zeta potential of the EV samples at pH 7.4 or 6.5 were measured using a Zetasizer 3000 instrument (Malvern Instruments, Malvern, UK) [3,30,37,38,39,40]. The morphologies of the EV samples at pH 7.4 or 6.5 were analyzed using a transmission electron microscope (Talos L120C, FEI, Hillsboro, OR, USA) [3,31,37,38].

### 2.7. In Vitro DOX Release Test 

The EV samples (equivalent to DOX of 100 μg/mL) in PBS (3 mL, 150 mM, pH 7.4) were added to a dialysis membrane (Spectra/Por^®^ MWCO 10 K) and immersed in fresh PBS (15 mL, 150 mM, pH 7.4 or 6.5) [3,30,31,40]. A DOX release test was conducted using a mechanical shaker (100 rpm) at 37 °C [3,30,31,40]. The external PBS of the dialysis membrane was extracted and replaced with fresh PBS at the specified time point. The amount of DOX released from the EV samples was measured using a fluorescence spectrofluorometer at λ_ex_ of 470 nm and λ_em_ of 592 [3,27]. 

### 2.8. Hemolysis Test

To determine the endosomolytic activity of the EV samples, a hemolysis test was conducted using red blood cells (RBCs) collected from BALB/c mice (7-week-old female) [31,41,42]. The RBC solutions (10^6^ cells/mL) at pH 7.4 or 6.5 were incubated with the EV samples (equivalent to EVs of 30 μg/mL, without DOX) at 37 °C for 1 h. The RBC solutions were centrifuged at 1500 *g* for 10 min at 4 °C and the supernatant was collected. The light absorbance (LA) value of the supernatant was measured using a spectrophotometer at a wavelength of 541 nm. A 0% (as a negative control) LA was acquired from a PBS-treated intact RBC solution and the 100% (as a positive control) of LA value was obtained from completely lysed RBC solution using 2 wt.% Triton X-100. The hemolysis (%) of each EV sample was determined as the LA of the RBC solution treated with each sample relative to the control LA value [31,41,42]. 

### 2.9. Cell Culture

Human breast carcinoma BT-474 cells, human neuroblastoma SK-N-MC cells, and human liver carcinoma Huh7 cells were purchased from the Korean Cell Line Bank. When the cells were grown as a monolayer (1 × 10^6^ cells/mL), they were harvested by trypsinization using a 0.25% (wt./vol.) trypsin/0.03% (wt./vol.) EDTA solution. Subsequently, the cells suspended in the RPMI-1640 or DMEM medium were seeded in well plates before cell test [37,38,39,40]. 

### 2.10. In Vitro Cellular UPTAKE test

BT-474, SK-N-MC, or Huh7 tumor cells were incubated with EV samples (equivalent to DOX of 5 μg/mL) or free DOX (5 μg/mL) for 4 h. After washing the cells using fresh PBS (150 mM, pH 7.4), the fluorescence intensity of the cells was determined using a FACSCaliburTM flow cytometer (FACS Canto II, Becton Dickinson, Franklin lakes, NJ, USA) [3,31,37,38,39]. In addition, to visualize the cellular uptake of the EV samples, BT-474 or SK-N-MC tumor cells were incubated with the EV samples for 4 h and then fixed using 3.7% formaldehyde in PBS. The fixed cells were monitored using a Nikon microscope equipped with a VNIR hyperspectral camera system (Cytoviva high-resolution adapter, Cytoviva, Auburn, AL, USA) [43,44,45]. For confocal microscopy analysis, the treated cells were stained with WGA-Alexa Fluor^®^ 488 and DAPI. The stained cells were then fixed using 3.7% formaldehyde in PBS and analyzed using a confocal laser scanning microscope (LSM710, Carl Zeiss, Oberkochen, Germany) [3,30,31].

### 2.11. In Vitro Cytotoxicity Test

To determine the cytotoxicity, BT-474, SK-N-MC, or Huh7 tumor cells were incubated with the EV samples (equivalent to DOX of 5 μg/mL) or free DOX (5 μg/mL) at pH 7.4 for 4 h. After washing the cells using fresh PBS (150 mM, pH 7.4), the treated cells were further incubated with fresh RPMI-1640 or DMEM medium at 37 °C for 24 h. CCK-8 assay was used to determine the cell viability [3,31,38,39,40]. In addition, the viability of the cells incubated with drug-free EVs for 24 h was measured using the CCK-8 assay to determine the original toxicity of the EVs and polymers [3,31,38,39,40].

### 2.12. In Vitro Cellular Uptake Test of EV Blends

BTEVs (with fluorescent Ce6 dye incorporation) and SKEVs (with fluorescent FITC dye incorporation) were used to visualize the cellular uptake of the EV blends. Here, Ce6 dye or FITC dye was incorporated into the EVs through sonication [3,30,36]. Briefly, Ce6 dye (200 μg) or FITC dye (200 μg) dissolved in DMSO (0.1 mL) were mixed with EVs (200 μg) suspended in PBS (10 mL, 150 mM, pH 7.4) at 25 °C; subsequently, the solution was sonicated using a tip sonicator, vcx-130 with cv-18 (Sonics, Newtown, CT, USA) with 30% amplitude for 30 s. The obtained solution was incubated at 37 °C for 60 min to recover the EVs. The filtration method using 0.22 μm membrane was performed to remove Ce6 or FITC aggregates. Ultracentrifugation at 100,000 *g* at 4 °C for 70 min was performed to remove free Ce6 or FITC. The measured weight of the Ce6 or FITC dye incorporated in the EVs was 1–2 wt.%, as evaluated using a fluorescence spectrofluorometer. The obtained EV blends [HDEA@BTEVs/HDEA@SKEVs (50/50 wt.%)] (equivalent to EVs of 30 μg/mL, without DOX) were added to the tumor cells seeded on coverslips in a culture plate at 37 °C for 4 h. After washing the cells using fresh PBS (150 mM, pH 7.4), the treated cells were fixed using 3.7% formaldehyde in PBS. The fixed cells were analyzed using a confocal laser scanning microscope (LSM710, Carl Zeiss, Oberkochen, Germany) [3,30,31].

### 2.13. In Vitro Cytotoxicity Test of EV Blends

The mixed tumor cells (BT-474/SK-N-MC = 50:50 ratio of the number of cells) were prepared before the cell test and incubated with EV blends [HDEA@BTEVs/HDEA@SKEVs (50/50 wt.%)] (equivalent to DOX of 5 μg/mL), HDEA@BTEVs (equivalent to DOX of 5 μg/mL), or HDEA@SKEVs (equivalent to DOX of 5 μg/mL) for 4 h at 37 °C. After washing the cells using fresh PBS (150 mM, pH 7.4), the treated cells were additionally cultured with fresh RPMI-1640/DMEM mixed medium (50/50 vol.%) at 37 °C for 24 h. The CCK-8 assay was used to determine the cell viability [3,31,38,39,40].

### 2.14. Statistics

All the experimental results were analyzed using Student’s t-test or ANOVA at a significance level of *p* < 0.01 (**) [37].

## 3. Results and Discussion

### 3.1. Characterization of EV Samples

To fabricate pH-sensitive EV blends for targeting heterogeneous tumor cells, we first harvested EVs (BTEVs from BT-474 tumor cells and SKEVs from SK-N-MC tumor cells). The harvested BTEVs and SKEVs were almost spherical, as shown in the TEM image (Figure S3a). Subsequently, these EVs (BTEVs and SKEVs) were identified to exhibit specific protein expressions (Figure S3b). In particular, TSG 101, a conventional marker for EVs, was highly expressed in all EVs [46,47,48]. The estrogen receptor alpha (ER-α) was significantly detected in the SKEVs, whereas the tau protein (Tau) was only detected in the BTEVs (Figure S3b) [49,50,51,52]. Next, we incorporated pH-sensitive polymers (HDEA) and an antitumor model drug (DOX) to the EVs through sonication, as described in the experimental methods section. Finally, we obtained HDEA-anchored BTEVs (HDEA@BTEVs) or HDEA-anchored SKEVs (HDEA@SKEVs). HDOC@BTEVs and HDOC@SKEVs were prepared as pH-insensitive EVs to evaluate the pH-sensitive properties of HDEA@BTEVs and HDEA@SKEVs, respectively. The loading content of HDEA or HDOC in the EV samples was 29–31 wt.%, and the loading content of DOX in the EV samples was 12–16 wt.% (data not shown). Next, we prepared EV blends by physically mixing HDEA@BTEVs and HDEA@SKEVs (a weight ratio of 50/50) in PBS (pH 7.4).

**Figure 1:**
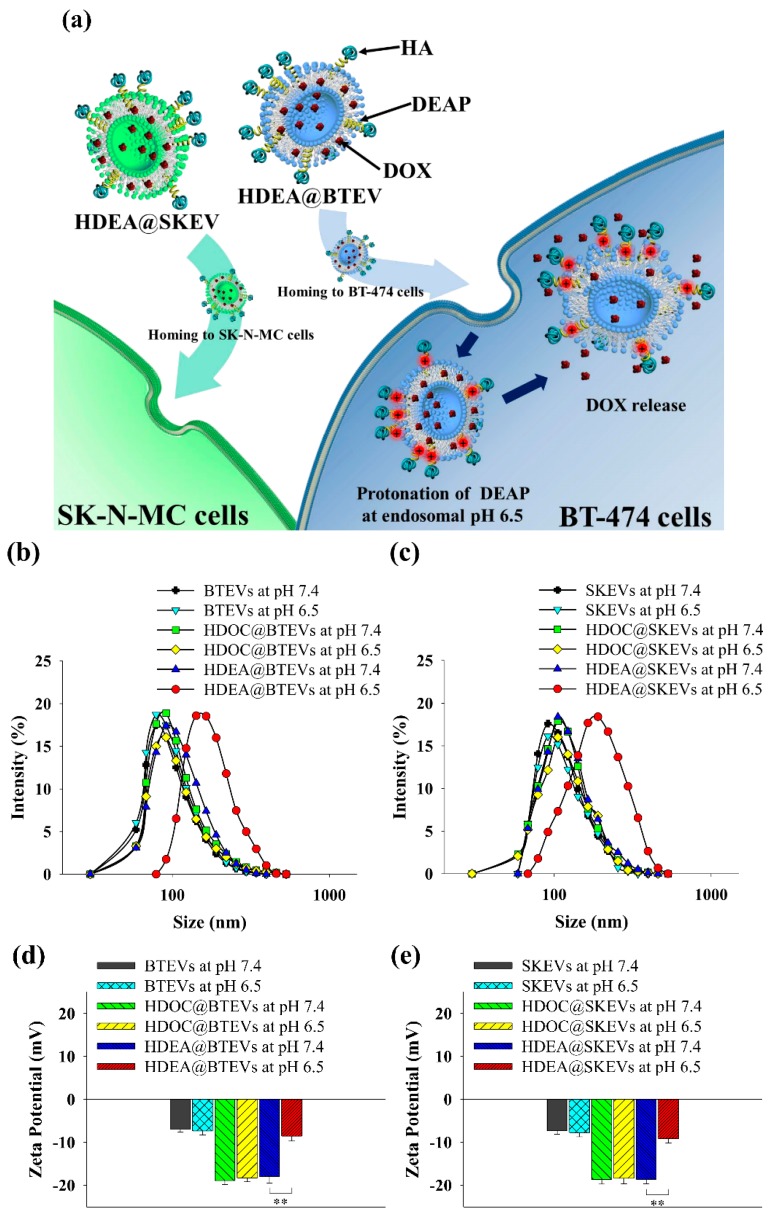

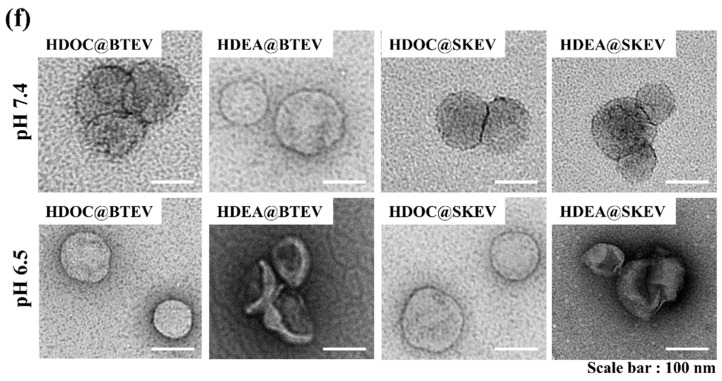
(**a**) Schematic illustration of tumor-homing pH-sensitive extracellular vesicles (EVs). Particle size distribution of (**b**) BTEVs and (**c**) SKEVs at pH 7.4 or 6.5. Zeta potential changes of (**d**) BTEVs and (**e**) SKEVs at pH 7.4 or 6.5 (*n* = 3, as multiple experiments, ** *p* < 0.01 compared to EVs at pH 7.4). (**f**) TEM images of EVs at pH 7.4 or 6.5.

As shown in Figure 1a, we expect the EV blends to home to their parent cells owing to the EVs’ homing ability [4,11,12,13,14,15,16,17]. Here, the DEAP moieties in the EVs can be protonated at the endosomal pH 6.5, inducing EV destabilization and accelerating the release of encapsulated DOX [3,23,24,25,26,27,28,29,30,31].

Figure 1b,c shows that the average particle sizes of intact EVs and the EV samples ranged from 105 to 120 nm at pH 7.4. However, the particle size of the HDEA@BTEVs and HDEA@SKEVs increased from 105 nm at pH 7.4 to 200 nm at endosomal pH 6.5 (Figure 1b,c), likely owing to the destabilized membrane of EVs due to the protonated DEAP [3,30,31]. By contrast, intact BTEVs, intact SKEVs, HDOC@BTEVs, and HDOC@SKEVs indicated no significant difference in particle size when the pH of the solution was reduced to pH 6.5; this could be due to the absence of pH-sensitive polymers (HDEA). In addition, as the pH of the solution decreased from 7.4 to 6.5, the zeta potentials of the HDEA@BTEVs and HDEA@SKEVs increased from −18.3 and −18.6 mV to −8.6 mV and −9.2 mV, respectively (Figure 1d,e). It was assumed that the protonated DEAP at pH 6.5 elevated the zeta potential of the HDEA@BTEVs and HDEA@SKEVs [3,30,31]. However, the zeta potential of intact BTEVs, intact SKEVs, HDOC@BTEVs, and HDOC@SKEVs indicated no significant difference at pH 7.4 and 6.5. Furthermore, the morphological images obtained from TEM reveal that almost spherical HDEA@BTEVs and HDEA@SKEVs at pH 7.4 were destabilized at pH 6.5, and that their structures were partially cracked (Figure 1f). However, the HDOC@BTEVs and HDOC@SKEVs did not show any noticeable changes between pH 7.4 and 6.5. These results demonstrated that pH-sensitive DEAP in the HDEA@BTEVs and HDEA@SKEVs mediated the destabilization of the EVs structure, owing to the protonation of HDEA [3,30,31] at endosomal pH 6.5. 

### 3.2. pH-Triggered DOX Release

**Figure 2:**
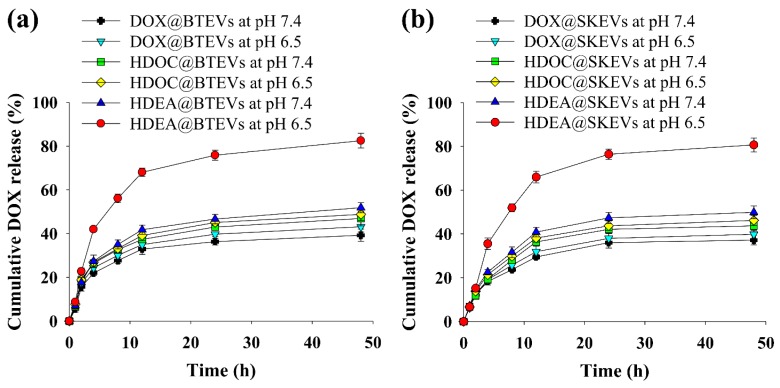
Cumulative doxorubicin hydrochloride (DOX) release from (**a**) BTEVs and (**b**) SKEVs at pH 7.4 or 6.5 in 48 h (*n* = 3, as multiple experiments).

The DOX release profile of the EV samples was monitored at pH 7.4 or 6.5 (Figure 2). At pH 7.4, all the EV samples released DOX gradually, and no significant differences were observed in the DOX release rates. However, at pH 6.5, the HDEA@BTEVs and HDEA@SKEVs exhibited a significant increase in the DOX release rates. Specifically, in 48 h, they released approximately 80–85 wt.% of the encapsulated DOX. In addition, regardless of pH change, the DOX@BTEVs, HDOC@BTEVs, DOX@SKEVs, and HDOC@SKEVs showed a passive DOX release of 40–45 wt.%. These results indicate that the HDEA@BTEVs and HDEA@SKEVs recognized a slight pH change, resulting in an accelerated DOX release at pH 6.5.

### 3.3. Endosomolytic Activity Test

**Figure 3:**
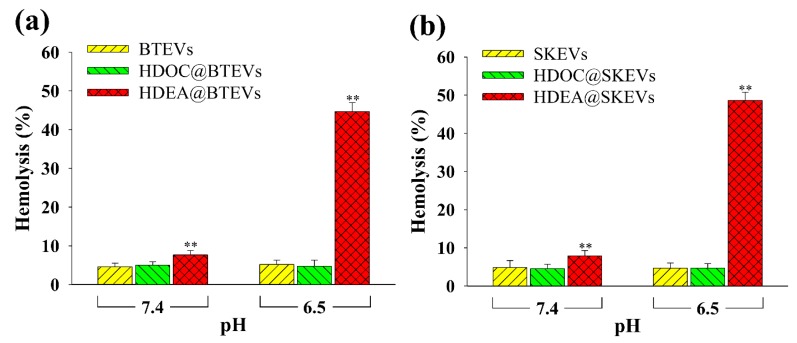
Hemolysis activities of (**a**) BTEVs and (**b**) SKEVs (*n* = 3, as multiple experiments, ** *p* < 0.01 compared with the control EVs).

To evaluate the endosomolytic activity of EV samples, we performed a hemolysis test using RBCs with an endosomal-like membrane (Figure 3). At pH 7.4, intact EVs and all the EV samples exhibited negligible hemolytic activity. However, in response to endosomal pH 6.5, the hemolytic activities of the HDEA@BTEVs and HDEA@SKEVs increased significantly, likely owing to the proton sponge effect [3,30,31] of the protonated DEAP. By contrast, intact BTEVs, HDOC@BTEVs, intact SKEVs, and HDOC@SKEVs indicated no significant difference in hemolytic activity at pH 6.5. These results indicate that the endosomolytic activity of the HDEA@BTEVs and HDEA@SKEVs facilitated the cytosolic release of drugs in the tumor cells. 

### 3.4. In Vitro Cellular Uptake and Cytotoxicity of EV Blends

**Figure 4:**
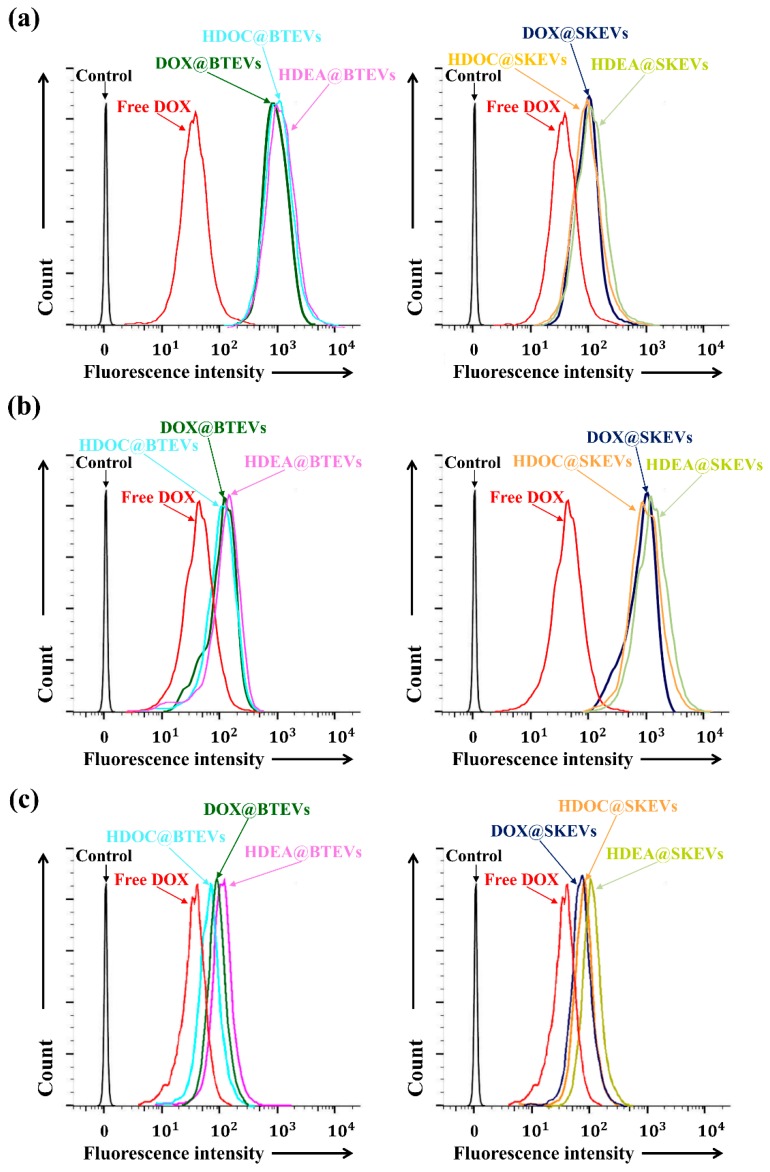
Flow cytometry profiles of (**a**) BT-474, (**b**) SK-N-MC and (**c**) Huh7 cells treated with free DOX (5 μg/mL) or EVs (equivalent to DOX of 5 μg/mL) for 4 h incubation at 37 °C.

We verified the cellular uptake behaviors of EV samples for tumor cells (BT-474, SK-N-MC, and Huh7 cells) using a FACSCaliburTM flow cytometer [3,31,37,38,39]. Figure 4 shows the quantitative results of the cellular uptake of EV samples in tumor cells (BT-474, SK-N-MC, and Huh7). The average fluorescence intensity of BTEVs samples (HDEA@BTEVs, HDOC@BTEVs, and DOX@BTEVs) in BT-474 tumor cells was ~1.4 × 10^3^; however, those of the SKEV samples (HDEA@SKEVs, HDOC@SKEVs, and DOX@SKEVs) and free DOX were ~1.2 × 10^2^ and ~45 (Figure 4a), respectively. Consequently, the uptake of the BTEV samples by the BT-474 tumor cells increased significantly, compared with that of the SKEV samples and free DOX. Meanwhile, as shown in Figure 4b, the uptake of the SKEV samples by the SK-N-MC tumor cells increased, compared with that of the BTEV samples and free DOX. The average fluorescence intensity of the SKEV samples in SK-N-MC tumor cells was ~1.6 × 10^3^; however, those of the BTEV samples and free DOX were ~1.5× 10^2^ and ~57, respectively. However, the uptake of all the EV samples in the Huh7 tumor cells was extremely low and did not exhibit a noticeable difference between all samples (Figure 4c). In addition, the average fluorescence intensity of all EV samples in the Huh7 tumor cells was ~1.1 × 10^2^. Overall, these results indicate that EVs with homing ability were efficiently endocytosed to their parent tumor cells [4,11,12,13,14,15,16,17].

**Figure 5:**
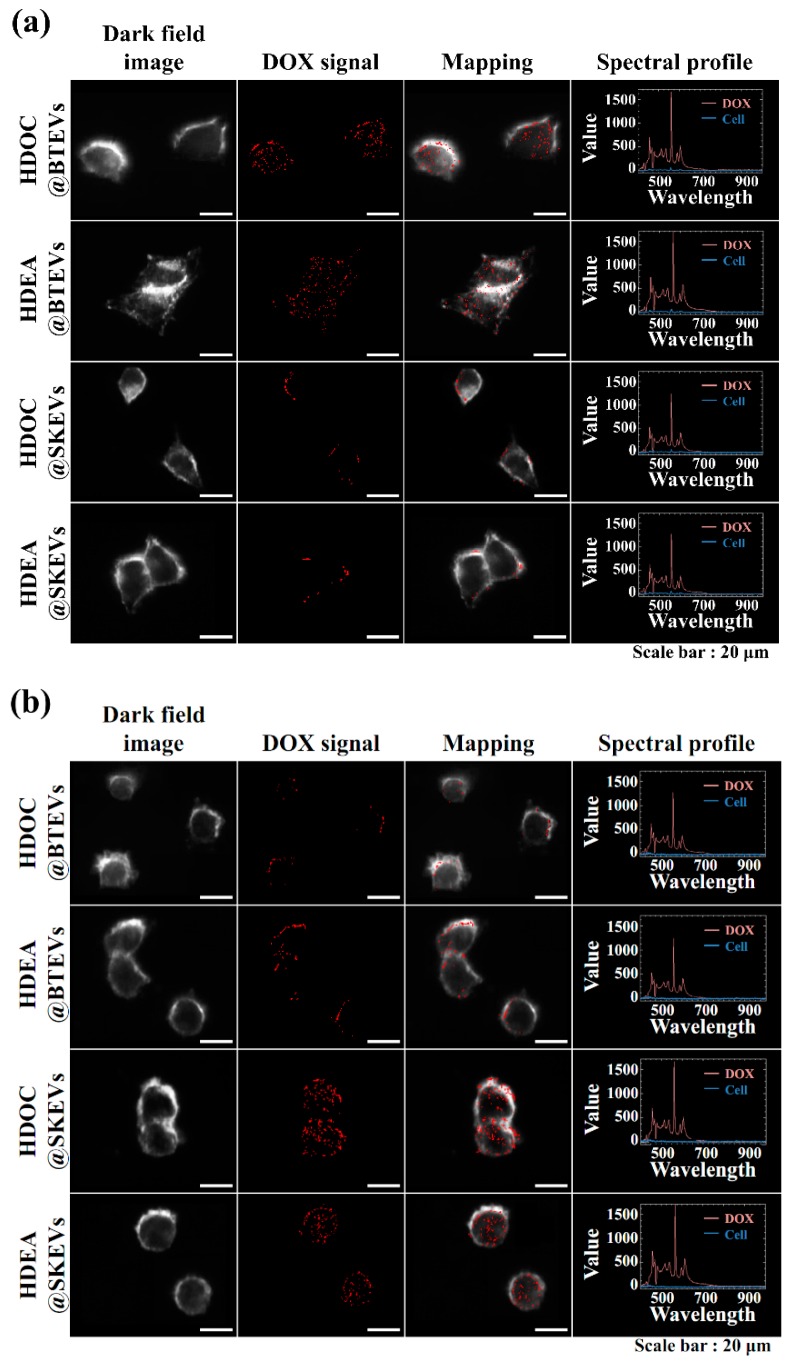
Hyperspectral images of (**a**) BT-474 and (**b**) SK-N-MC cells treated with EVs (equivalent to DOX of 5 μg/mL) for 4 h incubation at 37 °C.

**Figure 6:**
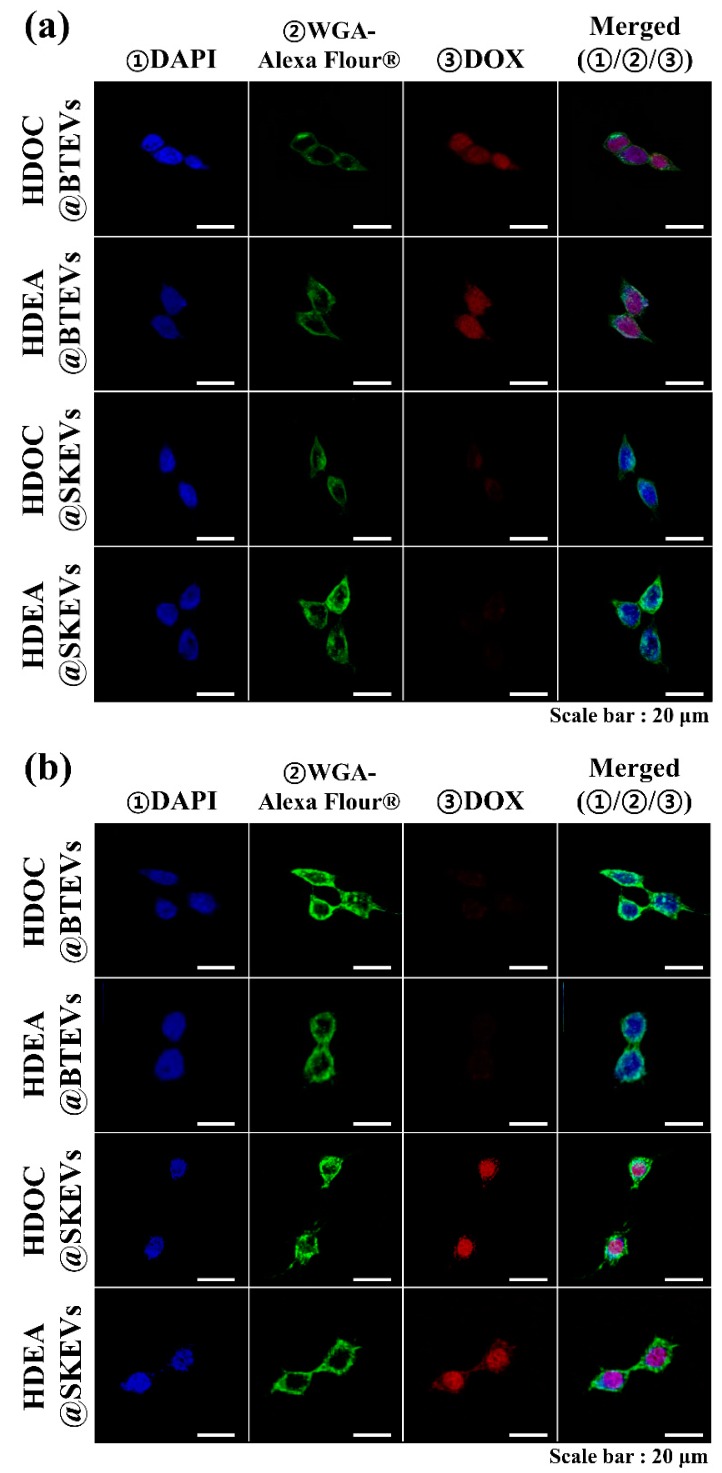
Confocal images of (**a**) BT-474 and (**b**) SK-N-MC cells treated with EVs (equivalent to DOX of 5 μg/mL) for 4 h incubation at 37 °C. The cells were stained using 4′,6-diamidino-2-phenylindole dihydrochloride (DAPI) and Wheat Germ Agglutinin Alexa Fluor^®^ 488 Conjugate (WGA-Alexa Fluor^®^488).

To further evaluate the internalization of EVs to their parent tumor cells, we also performed in vitro cell imaging studies using a VNIR hyperspectral camera system and a confocal laser scanning microscope [3,30,31,43,44,45]. The hyperspectral images shows that the DOX (present in EV samples) signal of BT-474 tumor cells treated using BTEV samples (HDEA@BTEVs and HDOC@BTEVs) was higher than that of BT-474 tumor cells treated with SKEV samples (HDEA@SKEVs and HDOC@SKEVs) (Figure 5a), whereas the DOX signal of SK-N-MC tumor cells treated using SKEV samples was higher than that of SK-N-MC tumor cells treated with BTEV samples (Figure 5b). Figure 6 shows confocal images of BT-474 and SK-N-MC tumor cells treated using EV samples. The treated cells were stained using DAPI and WGA-Alexa Fluor^®^ 488 to visualize the cell nuclei and membranes [3,30,31]. The confocal images revealed that the BTEV samples were actively internalized to BT-474 tumor cells, although the SKEV samples were ineffective in interacting with BT-474 tumor cells (Figure 6a). Similarly, the SKEVs samples were efficiently internalized to SK-N-MC tumor cells, whereas the BTEV samples were poorly internalized to SK-N-MC tumor cells (Figure 6b). These results support the homing ability of EVs to their parent tumor cells, which is consistent with the results shown in Figure 4.

**Figure 7:**
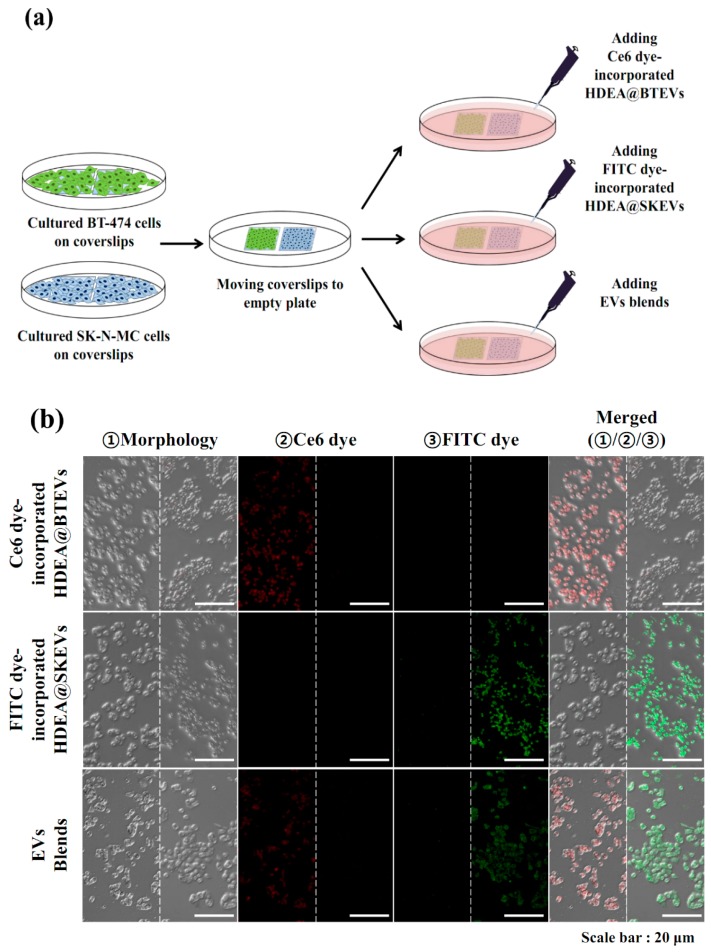

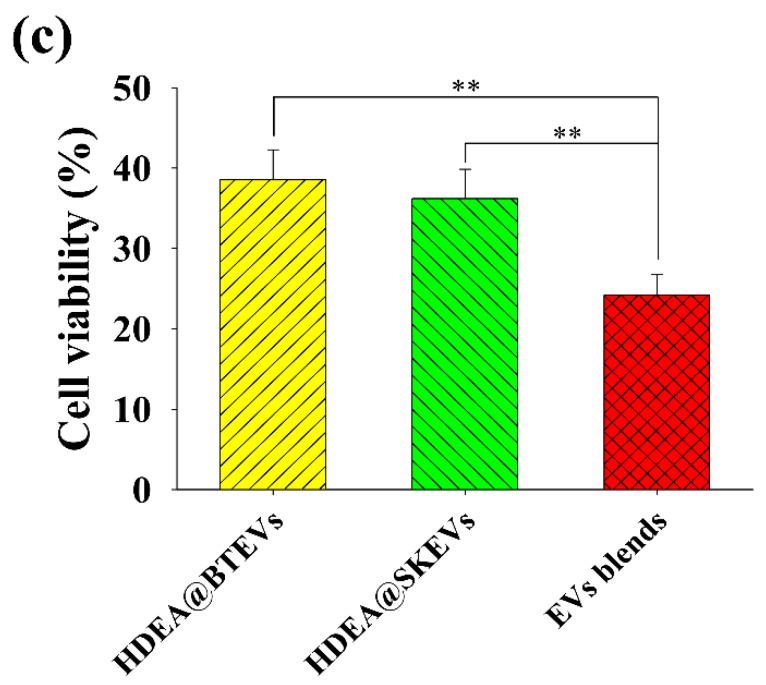
(**a**) Schematic illustration of in vitro experiments using Ce6 dye-incorporated HDEA@BTEVs, FITC dye-incorporated HDEA@SKEVs, and EV blends [Ce6 dye-incorporated HDEA@BTEVs/FITC dye-incorporated HDEA@SKEVs (50:50 wt.%)]. (**b**) Confocal images of BT-474 cells (left) and SK-N-MC cells (right) treated with Ce6 dye-incorporated HDEA@BTEVs, FITC dye-incorporated HDEA@SKEVs, or EV blends [Ce6 dye-incorporated HDEA@BTEVs/FITC dye-incorporated HDEA@SKEVs (50/50 wt.%)] (equivalent to EVs of 30 μg/mL) for 4 h incubation at 37 °C. (**c**) Cell viability determined by CCK-8 assay of tumor cells treated with EVs or EV blends [HDEA@BTEVs/HDEA@SKEVs (50/50 wt.%)] (equivalent to DOX of 5 μg/mL) for 4 h incubation at 37 °C (*n* = 7, as multiple experiments, ** *p* < 0.01 compared with each EV sample).

To verify the cellular uptake of the EV blends for BT-474 and SK-N-MC tumor cells, we performed in vitro cell imaging studies using a confocal laser scanning microscope [3,30,31]. As shown in Figure 7a, BT-474 and SK-N-MC tumor cells were first cultured on coverslips. Subsequently, the coverslips were moved to empty cell-culture plates and then treated with each EV samples or the EV blends. As shown in Figure 7b, Ce6 dye-incorporated HDEA@BTEVs displayed strong Ce6 fluorescence in BT-474 tumor cells, although the fluorescence intensity of Ce6 was weak in SK-N-MC tumor cells. By contrast, the FITC dye-incorporated HDEA@SKEVs displayed a strong FITC fluorescence signal for SK-N-MC tumor cells, but a weak FITC fluorescence signal in BT-474 tumor cells. More importantly, the EV blends displayed a strong Ce6 fluorescence and a weak FITC fluorescence in BT-474 tumor cells, whereas a weak Ce6 fluorescence and a strong FITC fluorescence in SK-N-MC tumor cells. These results indicate that the EV blends were selectively internalized to each parent tumor cells.

Figure 7c shows the cytotoxicity of the HDEA@BTEVs (equivalent DOX concentration of 5 μg/mL), HDEA@SKEVs (equivalent DOX concentration of 5 μg/mL), and the EV blends (equivalent DOX concentration of 5 μg/mL) to the mixed BT-474 and SK-N-MC tumor cells (50:50, cell number ratio). The EV blends exhibited highly increased tumor cell death for mixed BT-474 and SK-N-MC tumor cells. However, the HDEA@BTEVs or HDEA@SKEVs resulted in fewer tumor cell deaths. It is assumed that the efficient uptake of the EV blends in both BT-474 and SK-N-MC tumor cells resulted in the significantly increased antitumor efficacy. 

In addition, to evaluate the cytotoxicity of the EV samples, BT-474, SK-N-MC, and Huh7 tumor cells were treated using free DOX and EV samples at the equivalent DOX concentration of 5 μg/mL. 

**Figure 8:**
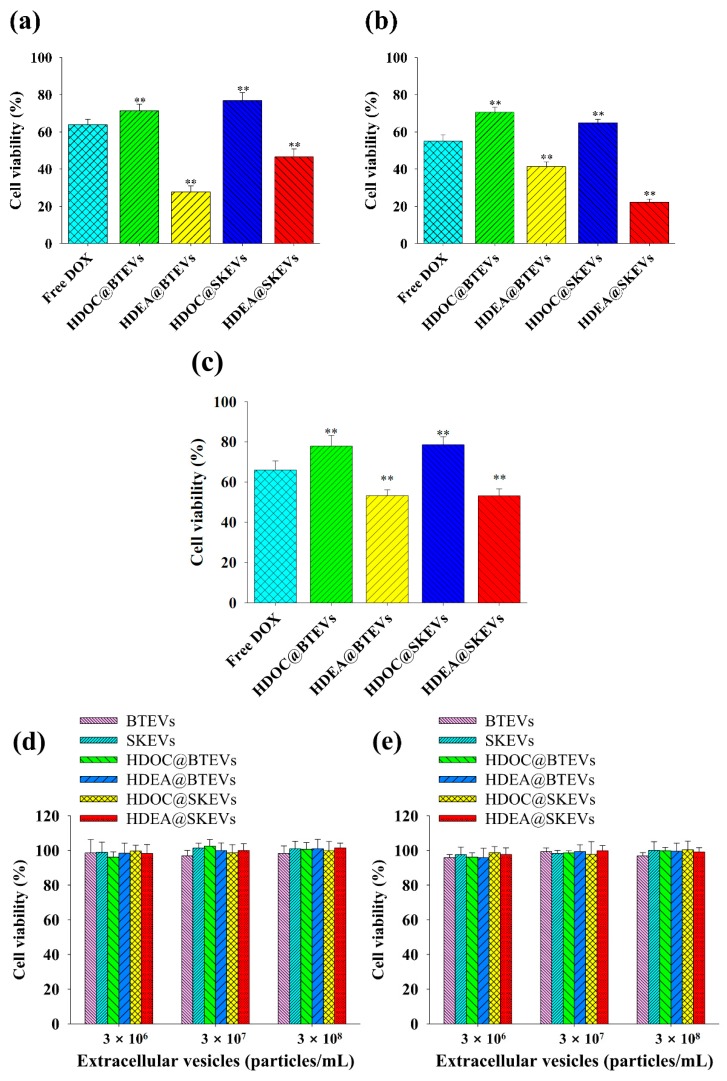
Cell viability determined by Cell Counting Kit-8 (CCK-8) assay of (**a**) BT-474, (**b**) SK-N-MC and (**c**) Huh7 cells treated with free DOX (5 μg/mL) or EVs (equivalent to DOX of 5 μg/mL) for 4 h incubation at 37 °C (*n* = 7, as multiple experiments), (** *p* < 0.01 compared with the free DOX). Cell viability determined by CCK-8 assay of (**d**) BT-474, (**e**) SK-N-MC, and (**f**) Huh7 cells treated with blank EVs (without DOX) for 24 h incubation at 37 °C (*n* = 7, as multiple experiments).

First, HDEA@BTEVs and HDOC@BTEVs exhibited relatively increased BT-474 tumor cell death compared with the SKEVs samples (HDEA@SKEVs and HDOC@SKEVs) (Figure 8a); this was likely owing to the homing ability of the BTEVs. However, the pH-insensitive HDOC@BTEVs resulted in a relatively reduced BT-474 tumor cell death, likely owing to the reduced DOX release (Figure 2). Similarly, the homing ability of the SKEVs resulted in the increased cell-cytotoxicity of the HDEA@SKEVs against SK-N-MC tumor cells (Figure 8b). However, Huh7 tumor cells treated with HDEA@BTEVs or HDEA@SKEVs did not exhibit noticeable levels of cell death. The low cellular uptake of the BTEV and SKEV samples in Huh7 tumor cells resulted in less cell-cytotoxicity for Huh7 tumor cells (Figure 8c). These results indicate that the homing ability of EVs to their parent tumor cells (Figure 4, Figure 5 and Figure 6) and the endosomolytic activity/endosomal pH-triggered DOX releasing property of HDEA-anchored EVs (Figure 2 and Figure 3) enabled a significantly improved tumor cell death. In addition, all EV samples without DOX showed negligible cytotoxicity up to 3 × 10^8^ particles/mL in 24 h for incubation with BT-474, SK-N-MC, and Huh7 tumor cells (Figure 8d–f), supporting their non-toxicity. 

## 4. Conclusions

In this study, tumor-homing pH-sensitive EV blends were fabricated using tumor-specific EVs (extracted from SK-N-MC and BT-474 tumor cells) and pH-sensitive HDEA. HDEA and DOX were incorporated in two different EVs through sonication; subsequently, they were blended in a weight ratio of 50:50. The EV blends, comprising two different EVs, can target two different parent tumor cells owing to the EVs’ homing ability. In addition, the pH-sensitive disruption of EVs owing to DEAP molecules promoted DOX release. Consequently, the EV blends killed the heterogeneous parent tumor cells effectively. Hence, we believe that EV blends with tumor cell targeting ability, the rapid release of DOX at the endosomal pH, and the enhanced antitumor effect may be novel strategies for treating various tumors. 

## Supplementary Materials

The following are available online at https://www.mdpi.com/1999-4923/12/4/372/s1, Figure S1. 1H-NMR analysis of HDEA. Figure S2. ^1^H-NMR analysis of HDOC. Figure S3. TEM analysis of intact (a) BTEV or SKEV. (b) Western blot analysis of the TSG 101 (tumor susceptibility gene 101), ER-α (estrogen receptor alpha), or Tau (tau protein) in BTEVs or SKEVs.
