# Supplementary material for: Tumor-Homing pH-Sensitive Extracellular Vesicles for Targeting Heterogeneous Tumors
Source: Pharmaceutics. 2020 Apr 17;12(4):372. doi: 10.3390/pharmaceutics12040372 (PMC7238000; doi:10.3390/pharmaceutics12040372)
Supplement: Supplementary file 1 [file pharmaceutics-12-00372-s001.pdf]

pharmaceutics

Supplementary Materials: Tumor-homing pH-sensitive Extracellular Vesicles for Targeting Heterogeneous Tumors

Jaeduk Park, Hyuk Lee, Yu Seok Youn, Kyung Taek Oh and Eun Seong Lee

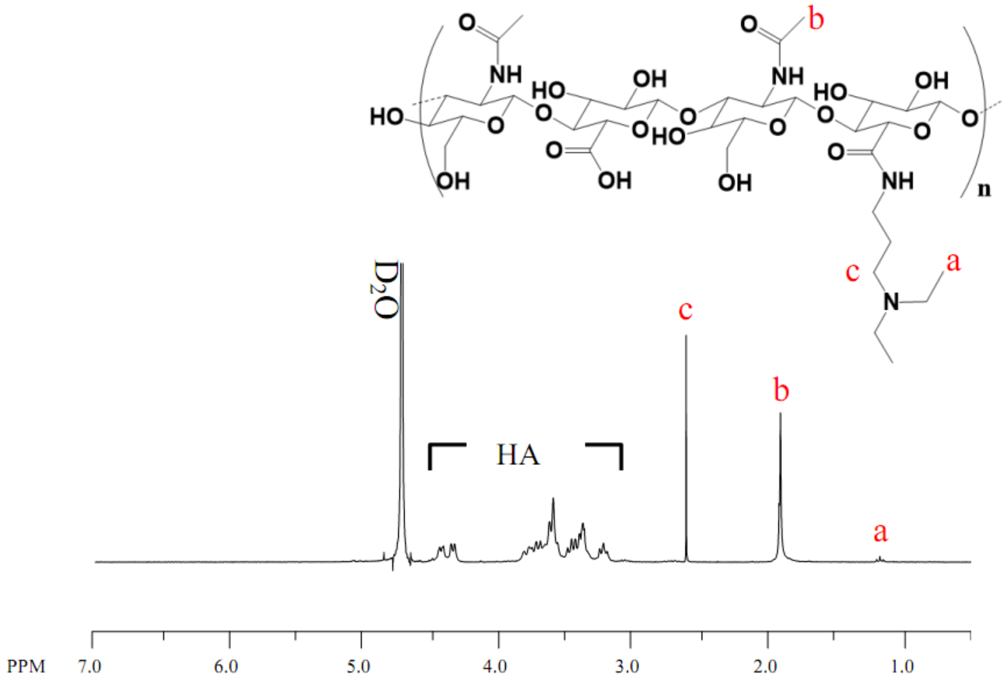

Figure 1. ^{1}H-NMR analysis of HDEA.

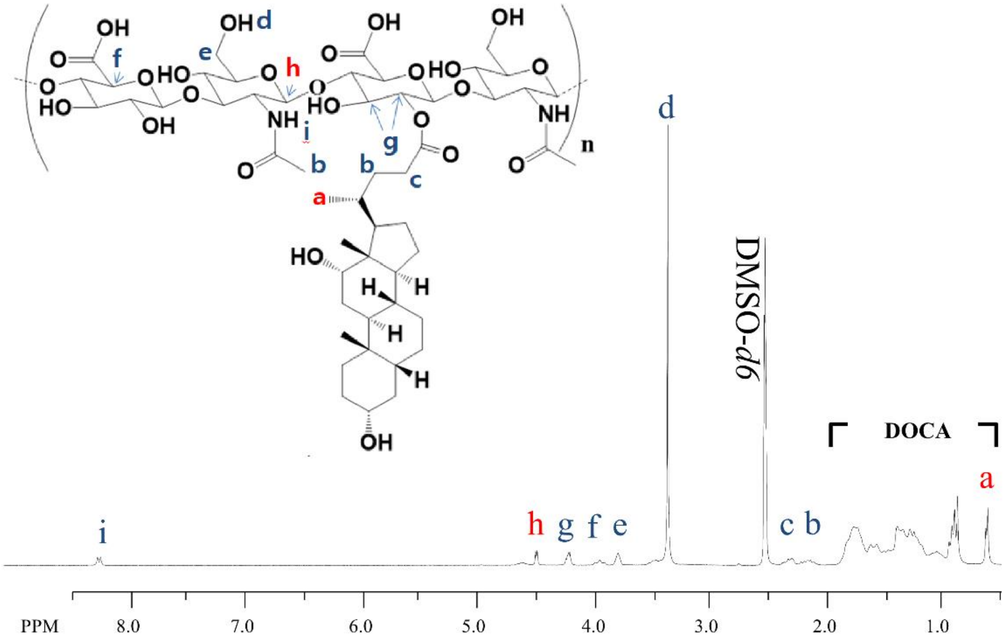

Figure S2. ^{1}H-NMR analysis of HDOC.

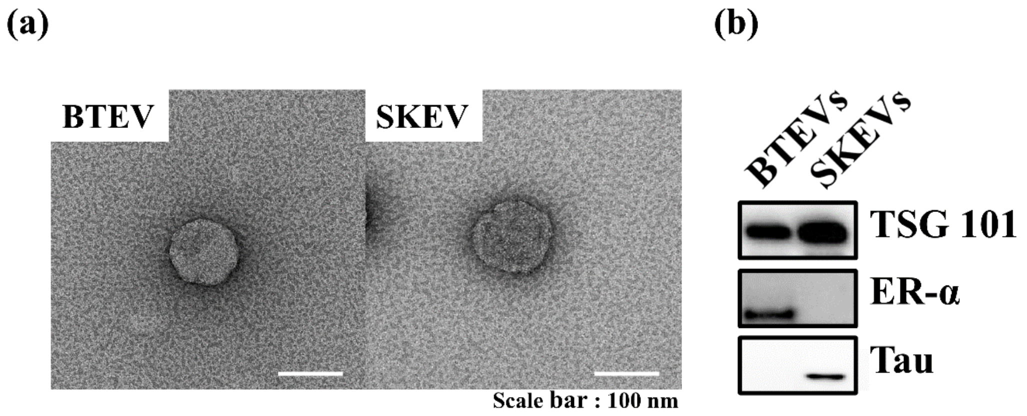

Figure S3. TEM analysis of intact (**a**) BTEV or SKEV. (**b**) Western blot analysis of the TSG 101 (tumor susceptibility gene 101), ER-α (estrogen receptor alpha), or Tau (tau protein) in BTEVs or SKEVs. To characterize unique protein expression on EVs, primary antibodies [rabbit monoclonal anti-TSG101 antibody (1 μg/mL, Abcam, Cambridge, UK), rabbit monoclonal anti-ER-α antibody (1 μg/mL, Abcam, Cambridge, UK) or rabbit monoclonal anti-Tau antibody (1 μg/mL, Abcam, Cambridge, UK)] and goat anti-rabbit IgG H&L secondary antibody (1 μg/mL, Abcam, Cambridge, UK) were used as primary and secondary antibodies [Chemiluminescent Reagent Kit (Abcam, Cambridge, UK)]. The experiments were performed using Chemi Doc MP System (Bio-Rad, Hercules, CA, USA).
